# Single-Cell Transcriptome Analysis Reveals Changes of Tumor Immune Microenvironment in Oral Squamous Cell Carcinoma After Chemotherapy

**DOI:** 10.3389/fcell.2022.914120

**Published:** 2022-06-17

**Authors:** Hao Song, Chao Lou, Jie Ma, Qiyu Gong, Zhuowei Tian, Yuanhe You, Guoxin Ren, Wei Guo, Yanan Wang, Kunyan He, Meng Xiao

**Affiliations:** ^1^ Department of Oral Maxillofacial-Head and Neck Oncology, Shanghai Ninth People’s Hospital, Shanghai Jiao Tong University School of Medicine, Shanghai, China; ^2^ Shanghai Key Laboratory of Stomatology & Shanghai Research Institute of Stomatology, National Clinical Research Center of Stomatology, Shanghai, China; ^3^ Department of Implant Dentistry, Shanghai Xuhui District Dental Center, Shanghai, China; ^4^ Shanghai Institute of Immunology, Faculty of Basic Medicine, School of Medicine, Shanghai Jiao Tong University, Shanghai, China; ^5^ Key Laboratory of Systems Biomedicine (Ministry of Education), Shanghai Center for Systems Biomedicine, Shanghai Jiao Tong University, Shanghai, China

**Keywords:** oral squamous cell carcinoma (OSCC), single-cell sequencing (scRNA-seq), introduction chemotherapy, tumor microenvironment, cancer-associated fibroblasts (CAFs)

## Abstract

Induction chemotherapy in oral squamous cell carcinoma is a controversial issue in clinical practice. To investigate the evolution of cancer cells and tumor microenvironment (TME) response to chemotherapy in oral squamous cell carcinoma, single-cell transcriptome analysis was performed in a post-chemotherapy squamous cell carcinoma located in oral cavity. The main cell types were identified based on gene expression patterns determined using dimensionality reduction and unsupervised cell clustering. Non-negative matrix factorization clustering of the gene expression of Cancer-associated fibroblasts (CAFs) and macrophages was performed. Kyoto Encyclopedia of Genes and Genomes pathway analyses and gene set enrichment analysis were performed to explore significant functional pathways. CellPhoneDB and NicheNet were used to detect the intercellular communication between cell types. CAFs were divided into “inflammatory CAFs,” “antigen-presenting CAFs” and “myofibroblastic CAFs.” Three classic subgroups of tumor-associated macrophages (TAMs) were detected, namely C1Q (+), FCN1 (+) and SPP1(+) TAMs. The inflammatory cytokine expression is elevated, and several molecular pathways, such as PI3K/Akt/mTORC1, TNF-α *via* NFκB, TGF-β, IL-6/JAK2/STAT3 and CXCL12/CXCR4 axis associated with epithelial-mesenchymal transition were enriched in TME. Also, CD74-MIF/COPA/APP interactions were expressed in TME of oral squamous cell carcinoma after chemotherapy. The results revealed the characteristics of TME in post-chemotherapy oral squamous cell carcinoma at single-cell transcriptome level, providing new insights and clues for further investigation.

## Introduction

Oral squamous cell carcinoma (OSCC) is a sub-category of head and neck squamous cell carcinoma (HNSCC), and is the most common malignant tumor in the oral cavity. Despite the use of radical surgical resection with reconstruction and postoperative radiotherapy or chemo-radiotherapy, the 5-year survival rate remains 50%–60% in locally advanced cases (Torre et al., 2015). The current NCCN guidelines for patients with resectable locally advanced OSCC recommend surgical management of the primary tumor and neck, followed by postoperative radiotherapy or chemo-radiotherapy, depending on the presence of intermediate- or high-risk features (National Comprehensive Cancer Network, 2022).

Induction chemotherapy (ICT) refers to the treatment of patients before surgical removal of the malignant tumor. ICT in OSCC is a controversial issue. It was reported that ICT reduced the risk of distant metastasis but had no impact on loco-regional control in HNSCC (Ma et al., 2012; Ma et al., 2013). It is still unclear whether ICT improves outcomes in patients with locally advanced OSCC. The front-line treatment for head and neck cancer involves docetaxel, cisplatin, fluorouracil, or a combination of these agents. Two clinical trials of ICT using the TPF regimen (docetaxel, cisplatin and fluorouracil) failed to demonstrate any benefit, with no significant impact of ICT on survival or loco-regional control when compared with up-front surgery (Licitra et al., 2003; Zhong et al., 2013). However, in a meta-analysis, ICT reduced loco-regional recurrence of OSCC (Lau et al., 2016). As previously reported in studies evaluating ICT for locally advanced HNSCC, superior outcomes were achieved based on both clinical and pathologic responses.

Assessment of molecular markers in bulk samples also enabled identification of patients most likely to benefit from ICT in OSCC. For example, p53 gene mutations have been strongly associated with lower response rates to PF induction (Temam et al., 2000; Perrone et al., 2010). Validation of p53 mutations and other biomarkers such as beta-tubulin and Bcl-xL as possible prognostic and predictive factors of response could ultimately facilitate the development of personalized induction treatments for resectable OSCC (Kumar et al., 2008; Cullen et al., 2009; Wu et al., 2012).

Further insights are needed before simplifying the loco-regional treatment based on major response to induction therapy. Strategies aimed to reduce the level of surgical resection or the need for radiotherapy or chemo-radiotherapy in place of surgery in patients with a favorable response to ICT deserve further investigation. Further studies are needed to elucidate the biology of malignant tumor cells and the tumor microenvironment (TME) after ICT.

High-throughput single-cell RNA sequencing (scRNA-seq) techniques have been used in tumor biology to elucidate the TME and the benefits of therapy in breast cancer, lung cancer and colon cancer (Kim et al., 2018; Maynard et al., 2020; Park et al., 2020). In 2017, Puram et al. (Puram et al., 2017) investigated the TME of OSCC via single-cell transcriptomic analysis using scRNA-seq for the first time. The investigators found a partial epithelial-mesenchymal transition (EMT) at the leading edge of the tumor nest regulated by the microenvironment. The expression of fibroblast subgroups in TME of OSCC was reproducibly detected across different tumors. Cancer-associated fibroblasts (CAFs) expressed notably higher numbers of ligands that correspond to receptors expressed by the cancer cells. These interactions included TGFB3-TGFBR2, FGF7-FGFR2, CXCL12-CXCR7.

Although studies revealed the single-cell transcriptome of treatment-naïve OSCC, the evolution of tumor cells as well as the TME response to chemotherapy remains unknown. In this study, scRNA-seq of the OSCC sample was performed after ICT for the first time. We reveal the characteristics of post-chemotherapy OSCC at the single-cell transcriptomic level.

## Materials and Methods

### Patient and Tumor Specimens

**FIGURE 1:**
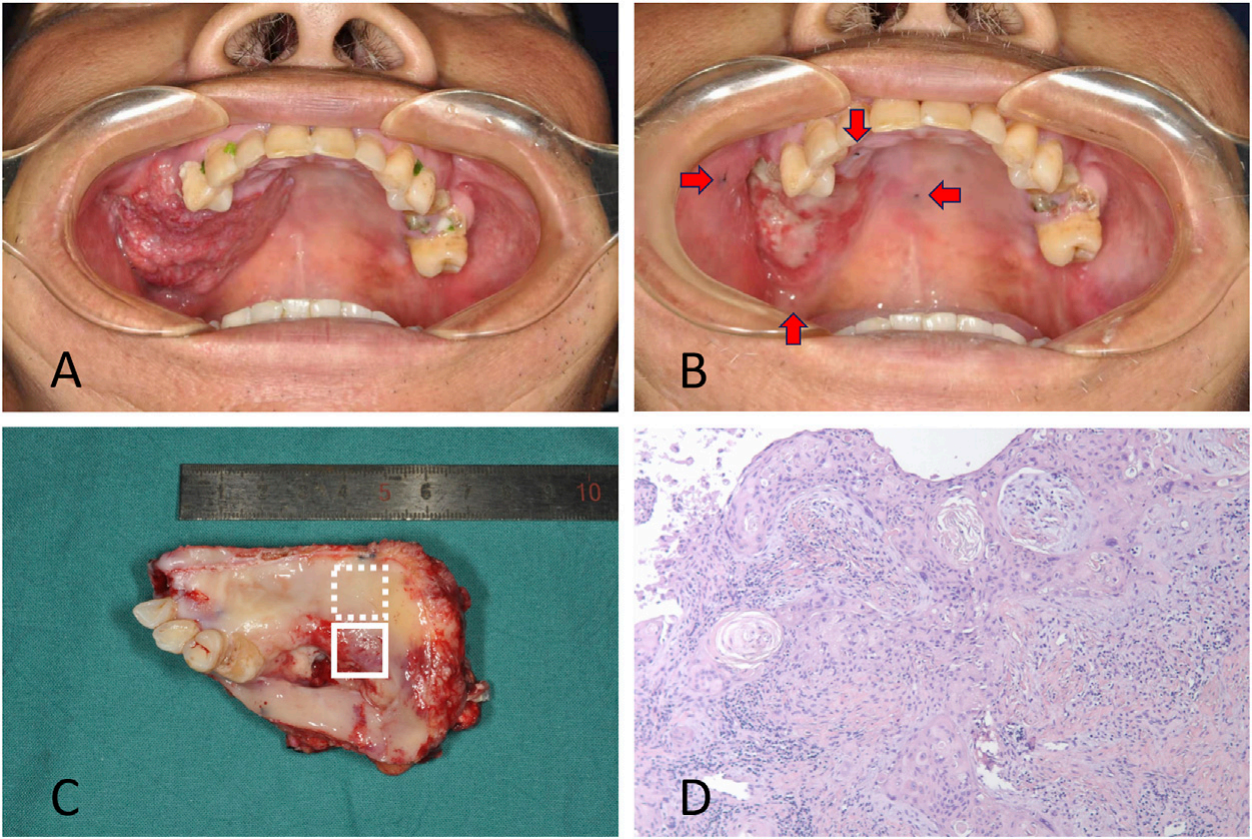
**(A)** The patient with squamous cell carcinoma located in gingiva of right maxilla. The primary lesion at the initial clinical evaluation before chemotherapy was shown. **(B)** The lesion after induction chemotherapy with a partial release. The marks showing the edges of the primary lesion made before chemotherapy were demonstrated by red arrows. **(C)** The resected tissue included the residual tumor (solid line box) and tumor bed (dotted line box). **(D)** Pathologic exam confirmed the residual tumor cells in the tissue.

Tumor and adjacent tissues were obtained from a patient diagnosed with OSCC in Shanghai Ninth People’s Hospital, Shanghai Jiao Tong University School of Medicine. The patient was a 50-year-old male with squamous cell carcinoma located in the gingiva of right maxilla. Samples were collected with the approval of the institutional ethics committee of Shanghai Ninth People’s Hospital. Written informed consent was obtained for the enrolled patient. The resection volume was planned considering the tumor extension at the initial clinical evaluation and the palpable edges of the primary lesion were marked before ICT, which were 0.5 cm away from the lesion (Figure 1A). The patient was treated with ICT. Chemotherapy consisted of docetaxel 75 mg/m^2^ and cisplatin 75 mg/m^2^ on day 1, followed by fluorouracil 750 mg/m^2^ daily as a 120-h continuous infusion on days 1 through 5. ICT was administered every 3 weeks for two cycles. The tumor responded to chemotherapy with a partial release (PR) according to RECIST 1.1 (Figure 1B). Radical resection of the primary lesion and neck dissection was performed after ICT. The safe margins were 1.0 cm away from the marks placed before ICT to ensure that the extent of surgery remained at the same level before chemotherapy (Figures 1C,D).

The study was conducted in accordance with the Declaration of Helsinki (as revised in 2013). The retrospective study was approved by the Institutional Research Ethics Committee of Shanghai Ninth People’s Hospital (SH9H-2020-TK471-1) and the informed consent was waived.

### Isolation of Single-Cell Suspension

OSCC and adjacent non-cancerous tissues were obtained from the operating room after surgical resection immediately at the Shanghai ninth people’s hospital and transferred to the laboratory in cold Hank’s balanced salt solution medium supplemented with 1% penicillin–streptomycin within 30 min. The extension of radical surgical resection was performed according to the initial clinical evaluation before ICT. The resected tissue included the residual tumor and tumor bed, which were sampled and analyzed separately. Tumor bed, where the original tumor has released, served as the control. Each sequencing tissue was cut into about 1 mm^3^ pieces and digested, followed by filtration of the cell suspension using a 70-μm diameter filter. Dissociated cells were pelleted and resuspended in phosphate-buffer saline. The single-cell suspension was placed on ice until cells were loaded onto the 10X Chromium Single Cell Platform (10X Genomics) at a concentration of 1,000 cells per μl (Single Cell 3′library and Gel Bead Kit v.3) as described in the manufacturer’s protocol. Generation of gel beads in emulsion (GEMs), barcoding, GEM-RT clean-up, complementary DNA amplification and library construction were performed as per the manufacturer’s protocol. Qubit was used for library quantification before pooling. The final library pool was sequenced on Illumina Nova6000 using 150-base-paired end reads.

### scRNA-Seq Data Quality Control (QC)

R (version 4.0.5, https://www.r-project.org/) and Seurat R package (version 4.0.1, https://satijalab.org/seurat/) were used for QC and data secondary analysis. Single cells were filtered for downstream analysis according to the following criteria: Cells expressing less than 500 or more than 4,500 genes (potential cell duplets) and gene expression not detected in fewer than 10 cells were trimmed from the library. The unique molecular identifier (UMI) count was less than 20,000, and the mitochondrial percentage was less than 15% of the total UMI count. After data normalization, variably expressed genes were normalized and scaled. The resulting gene expression was transformed into log space.

### Cell-type Clustering and Identification

Principal component analysis (PCA) was used to reduce the data dimensions of variably expressed genes. The closer the sample distance, the closer is the expression of the cell genes. Single-cell clustering was visualized using uniform manifold approximation and projection (UMAP) of the top 16 principal components of the PCA results with the largest variance explained (RunUMAP function, the default setting). With a resolution of 0.25, the cells were clustered using the FindClusters function and classified into different cell types. Differentially expressed genes (DEGs) in each cell type were identified using the FindAllMarkers function in Seurat R package (only.pos = T). To further characterize these cell types in each cluster, we used the automated annotation tool SingleR (Aran et al., 2019) and manually checked using known cell markers based on previous studies (Puram et al., 2017; Chen et al., 2020). To better understand CAFs and macrophages, non-negative matrix factorization (NMF) clustering of the gene expression of CAFs and macrophages was performed using the NMF R package (Gaujoux and Seoighe, 2010). NMF is a group of algorithms in multivariate analysis and linear algebra, and is widely used in various fields, including artificial intelligence, signal processing and bioinformatics. We performed NMF with the number of factors k = 3 for CAFs and k = 4 for macrophages. The DoHeatmap function of the Seurat package was used for producing the heatmaps.

### Comparing scRNA-Seq Data With Previous Data

The scRNA-seq dataset GSE103322 of OSCC samples from 18 different treatment naïve patients were from the NCBI gene expression omnibus (Puram et al., 2017). The functions of FindIntegrationAnchors and IntegrateData wrapped in the R package of Seurat were employed to merge our data with the data derived from the previous study and remove the batch effect based on standard procedure. UMAP plot was used to represent cells from samples derived from 19 different patients.

### Identification of Differential Gene Expression

Analysis of differential gene expression was performed after normalization and removal of the batch effects of total genes from specific clusters derived from different samples. The function of FindAllMarkers wrapped in the R package of Seurat was used to identify differentially expressed genes in each of the cell clusters compared with others. DEGs were detected with a false discovery rate (FDR) < 0.05 and abs (avg_log2FC) > 0.25.

### Analysis of Cell Function Using KEGG and GSEA

To investigate the underlying biological functions of DEGs, Kyoto Encyclopedia of Genes and Genomes (KEGG) pathway analyses were performed to explore significant functional pathways. The adjusted *p*-values were calculated using Benjamini and Hochberg method. The P.adjust value <0.05 was considered statistically significant. The “ggplot2” package was utilized to visualize KEGG pathways. Furthermore, gene set enrichment analysis (GSEA) was conducted using the R package clusterProfiler to elucidate the enriched pathways significantly altered between residual tumor and tumor bed (Yu et al., 2012).

### Complex Cell–Cell Communication Networks in the TME

To characterize the TME of OSCC after ICT, CellPhoneDB was used to detect the intercellular communication between cell types. CellPhoneDB is a publicly available repository of curated receptors and ligands and their interactions (Vento-Tormo et al., 2018). CellPhoneDB analysis was performed using the CellPhoneDB Python package. Ligand and receptor information used in analysis were retrieved from CellPhoneDB (https://www.cellphonedb.org) repository. This package searches for a ligand-receptor interaction and outputs multiple result files based on curated databases. Circle plots depicting the amount of interactions between cell types were drawn using the function of chordDiagram in the R package of circilize. log10 of *p* values and log2 mean expression was calculated and expressed on dotplots. Single-cell transcriptomic data of cells annotated as epithelial cells, CD4^+^ T cells, CD8^+^ T cells, B cells, dendritic cells, macrophages, mast cells, endothelial cells, follicular dendritic cells (FDCs), myofibroblasts and CAFs were fed into CellPhoneDB to perform the analysis of cell–cell interaction. Enriched receptor–ligand interactions between cell types were based on the receptor expression on a specific cell type and the expression of the corresponding ligand by another cell type. The most relevant cell type-specific interactions between ligands and receptors were identified. To further predict the potential ligand–receptor communication between cell types in TME, we used the NicheNet method (Browaeys et al., 2020). NicheNet is a computational modeling tool based on gene expression data used in models of cell-cell communication linking ligands to target genes. Contrary to CellPhoneDB, NicheNet analyzes gene regulatory effects of ligands because the prior knowledge exceeds ligand-receptor interactions and incorporates intracellular signaling and transcriptional regulation. Using cell type labeled in Seurat object, analysis was performed with the function of nichenet_seuratobj_aggregate in nichenetr package. The receiver cell type is specified as epithelial cells, whereas condition of interest is ‘residual tumor’. Nichenet heatmaps were plotted using nichenet$ligand_receptor_heatmap and nichenet$ligand_activity_target_heatmap.

## Results

### Single-Cell Profiling in OSCC After ICT

**FIGURE 2:**
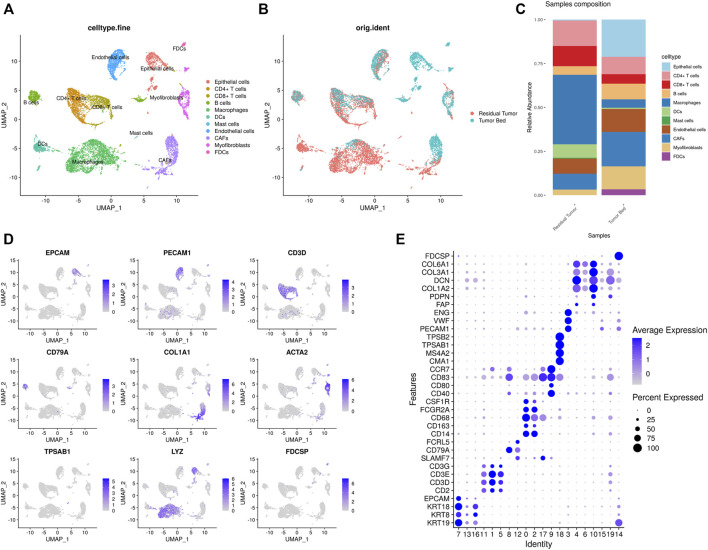
Single-cell transcriptomic map of OSCC after ICT **(A,B)**. **(A)** UMAP plot presentation of OSCC grouped by annotated cell types. **(B)** UMAP plot of original ident (from residual tumor or tumor bed). **(C)** The composition of cell types shown in residual tumor sample and tumor bed sample. **(D)** The expression of marker genes shown in feature plots. **(E)** Dot plots showing the maker genes of each cluster of OSCC.

In the present study, scRNA-seq identified a total of 12856 cells from residual tumor (8,326 cells) and tumor bed (4,530 cells). The main cell types were identified based on gene expression patterns determined using dimensionality reduction and unsupervised cell clustering with Seurat pipeline. Twenty clusters (cluster 0–19) were characterized. Based on the marker genes of each cell cluster, nine major cell types were identified, including epithelial cells (EPCAM, KRT8, KRT14, KRT17, KRT18, and KRT19); fibroblasts (FAP, PDPN, COL1A2, DCN, COL3A1, and COL6A1); T cells (CD2, CD3D, CD3E, and CD3G); B cells (SLAMF7, CD79A, and FCRL5); mast cells (CMA1, MS4A2, TPSAB1, and TPSB2); dendritic cells (CD40, CD80, CD83, and CCR7); macrophages (CD14, CD163, CD68, FCGR2A, and CSF1R); FDCs (FDCSP); and endothelial cells (PECAM1, VWF, and ENG). T cells were further subclustered into CD8^+^ T cells (CD8A, GZMK, CCL5, NKG7) and CD4^+^ T cells (IL7R, CD40LG, MAF), while fibroblasts were divided into CAFs (FAP, PDPN) and myofibroblasts (ACTA2, MYLK, MYL9). (Figure 2).

In the sample residual tumor, macrophages (3,293 cells, 39.6%), T cells (2,169, 26.1%) and fibroblasts (1,018, 12.2%) were the most abundant cell populations. In the tumor bed, fibroblasts (1,478, 32.6%), epithelial cells (963, 21.3%) and T cells (690, 15.2%) accounted for 69.1% of the cell population. Mast cells 39) were only detected in residual tumor, while FDCs 153) were only found in the tumor bed sample.

**FIGURE 3:**
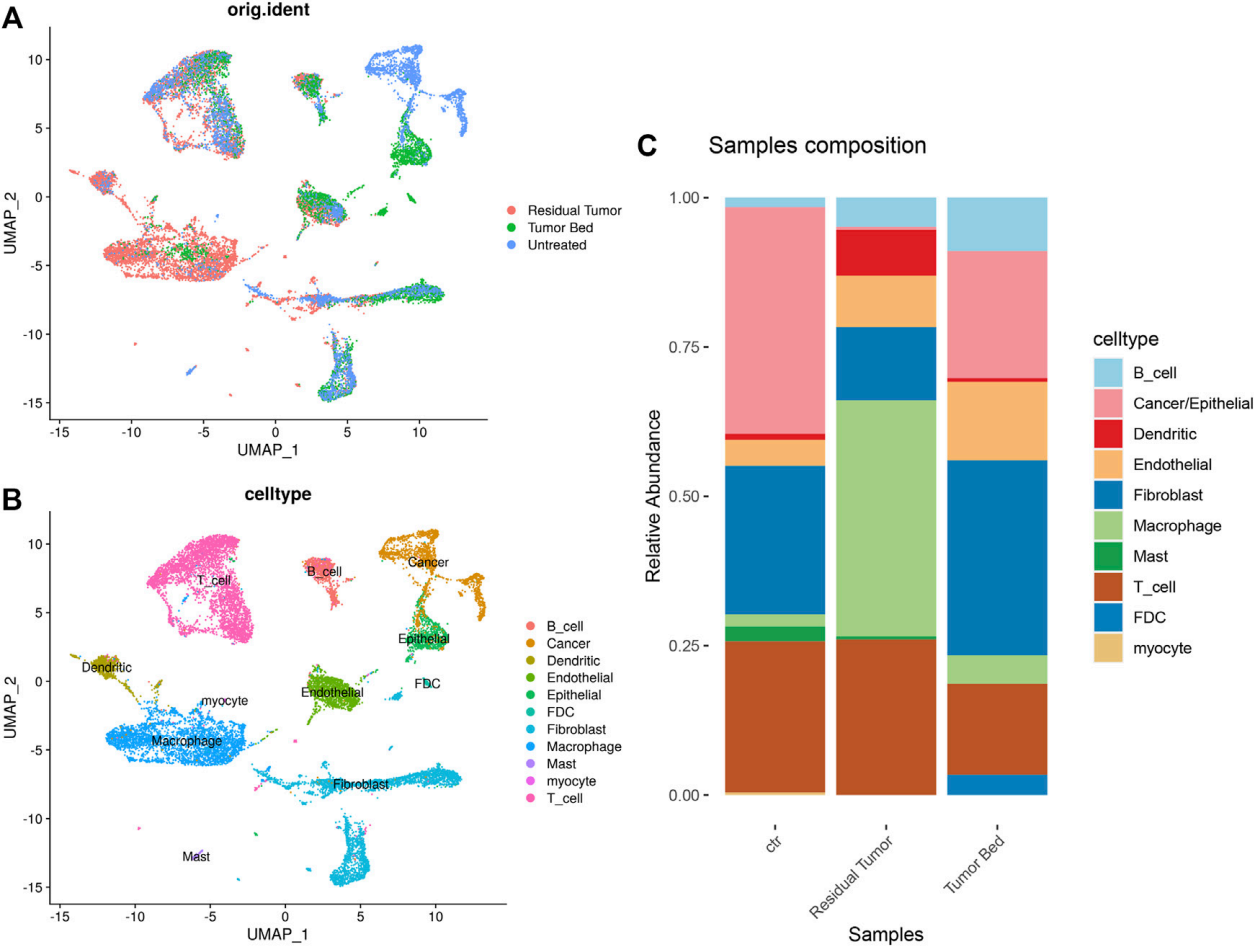
Single-cell transcriptomic map of merged data of OSCC sample containing post-chemotherapy tumor tissue and treatment naïve tumor. **(A)** UMAP plot of original ident (from residual tumor, tumor bed or untreated tumor). **(B)** UMAP plot presentation of OSCC grouped by annotated cell types. **(C)** The composition of cell types shown in treatment naïve OSCC, residual tumor and tumor bed.

In the combined data of our study and GSE103322, we used the annotations in the previous study. The cells were classified as ‘Cancer/Epithelial’, ‘Dendritic’, ‘Macrophage’, ‘Myocyte’, ‘B cell’, ‘T cell’, ‘Endothelial’, ‘Fibroblast’ and ‘Mast’. Cell clustering and annotation in our data were consistent with the previous study, which strongly supported the cellular annotation of the OSCC sample after chemotherapy (Figures 3A,B). As untreated OSCC tumor samples from 18 different patients served as control, the proportion of cells differed in residual tumor tissues. The proportion of epithelial cells was much lower in the residual tumor. The proportion of macrophages and dendritic cells were substantially higher in the residual tumor sample than in the untreated tumor. FDCs were only found in tumor bed, and myocytes were only detected in the untreated sample (Figure 3C).

### Subclustering and Differential Gene Profiles of CAFs and Macrophages

**FIGURE 4:**
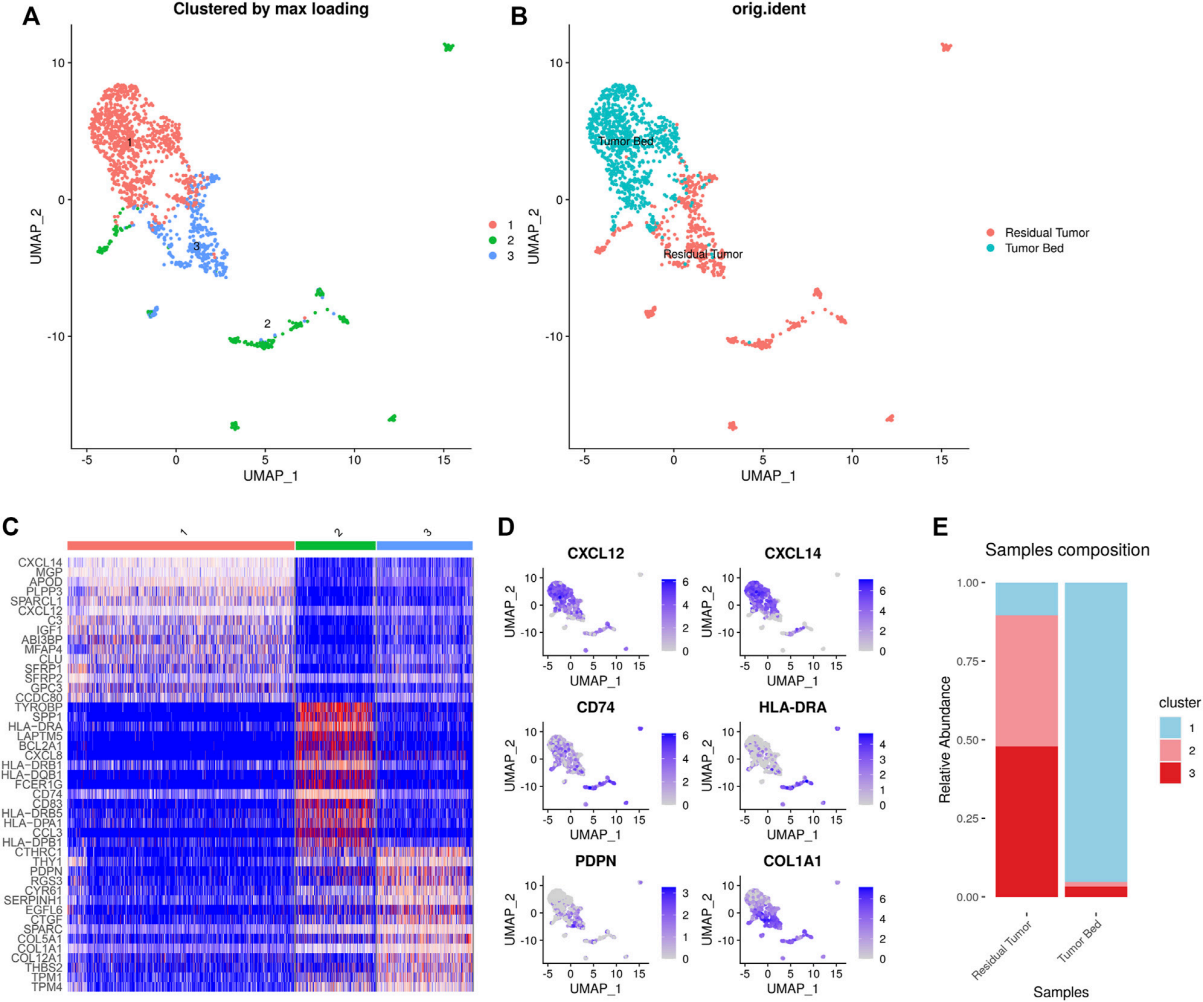
Heterogeneity of CAFs in OSCC after ICT. UMAP plot showing the subclusters divided using NMF **(A)** and the original ident **(B)**. Marker genes expression shown in heatmap. C1 CAFs expressed CXCL12, CXCL14, and IGF1. C2 CAFs expressed CD74 and MHC-Ⅱ. C3 CAFs expressed PDPN and COL1A1 **(C)**. Feature plots showing CXCL12, CXCL14, CD74, HLA-DRA, PDPN, COL1A1 expression in CAFs **(D)**. The proportion of subclusters in residual tumor and tumor bed. C1 CAFs were mainly enriched in tumor bed and C2 and C3 CAFs were enriched in residual tumor **(E)**.

NMF clustering of the gene expression of CAFs and macrophages was performed for subclustering. We detected 1,641 CAFs in the samples including 753 cells in the residual tumor and 888 cells located in the tumor bed. Three distinct subtypes of CAFs were identified. Cluster 1–3 (C1-C3) CAFs included 925, 326 and 390 cells, respectively. C1 CAFs were strongly enriched in tumor bed with 79 cells in residual tumor and 846 in tumor bed. C1 CAFs expressing CXCL12 were designated as inflammatory CAFs (iCAFs) in previous studies (Chen et al., 2021). C1 CAFs also expressed CXCL14 and IGF1. C2 and C3 CAFs were enriched in residual tumor, with 96.0% (313 cells) C2 CAFs located in residual tumor. C2 CAFs expressed CD74 and MHC-Ⅱ (such as HLA-DRA, HLA-DRB1, HLA-DQB1, HLA-DRB5, HLA-DPA1 and HLA-DPB1), which were reported as ‘antigen- presenting CAFs (apCAFs)’ (Elyada et al., 2019; Chen et al., 2021). Residual tumors contained 92.6% (361 cells) of C3 CAFs expressing PDPN and COL1A1, which was consistent with ‘myofibroblastic CAFs (myCAFs)’ (Chen et al., 2021) (Figure 4).

**FIGURE 5:**
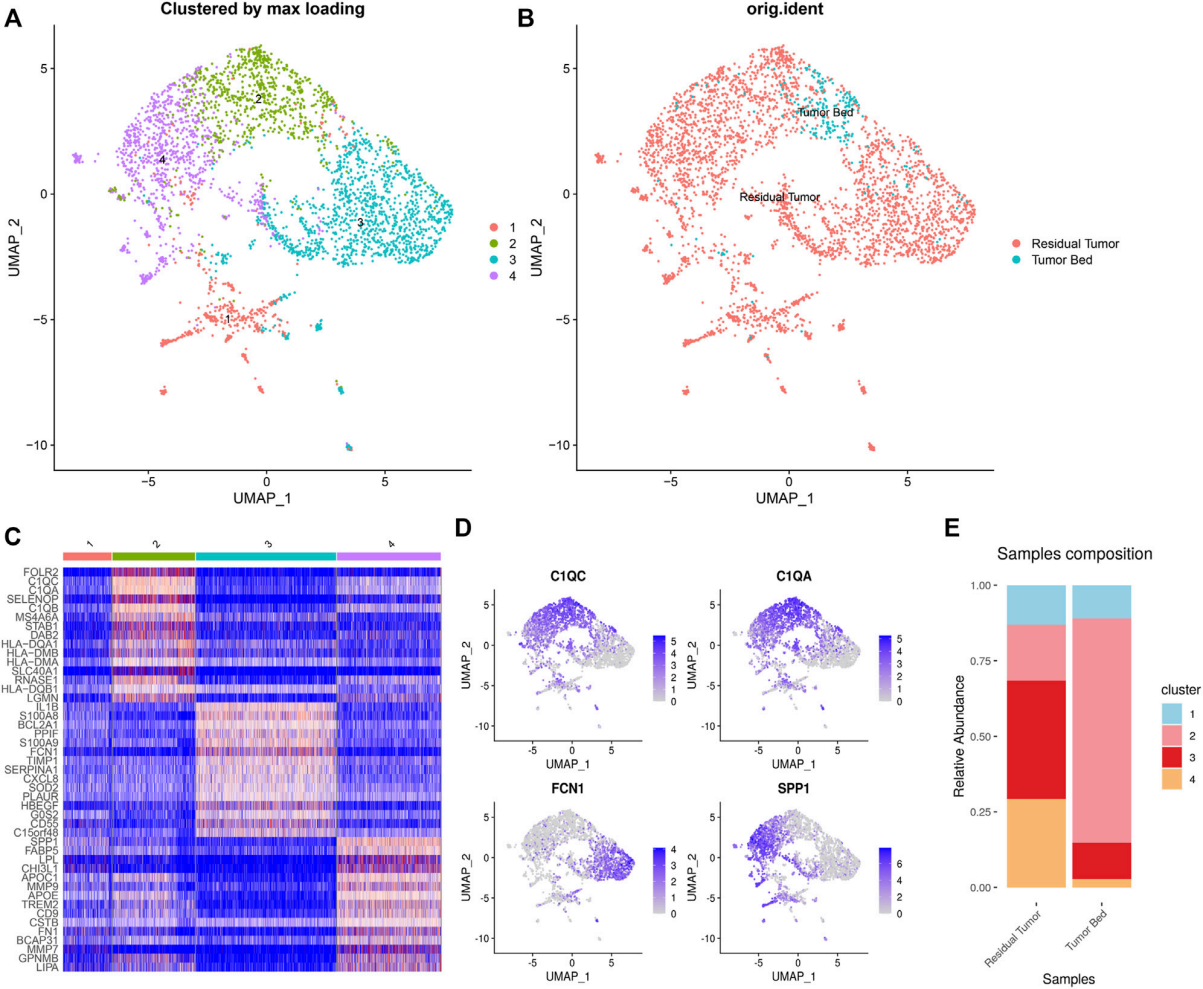
Heterogeneity of macrophages in OSCC after ICT. UMAP plot showing the subclusters divided using NMF **(A)** and the original ident **(B)**. Marker genes expression shown in heatmap, highlighting three classic subgroups of TAMs (C2–4). C2 TAMs expressed C1Qs. C3 CAFs expressing FCN1. C4 CAFs expressed SPP1 **(C)**. Feature plots showing C1QC, C1QA, FCN1, SPP1 expression in TAMs **(D)**. The proportion of subclusters in residual tumor and tumor bed **(E)**.

Of the 3,510 macrophages in total, 92.8% (3,293 cells) were located in the residual tumor. Four clusters of tumor-associated macrophages (C1-C4 TAMs) included 454, 772, 1,314, and 970 cells, respectively. The residual tumor contains 94.7% (430 cells) of C1 TAMs, which express CD7, CD3D and CD3E. The residual tumor contained 79.1% (611 cells) of C2 TAMs, while the tumor bed contained 161 cells. C2 TAMs expressed C1Qs (C1QC, C1QA and C1QB). The residual tumor contained 98.0% (1,288 cells) C3 TAMs, which expressed FCN1. C4 TAMs (964 cells in residual tumor) express SPP1, TREM2 and APOE (Figure 5).

### Pathway Analysis Using KEGG and GSEA

**FIGURE 6:**
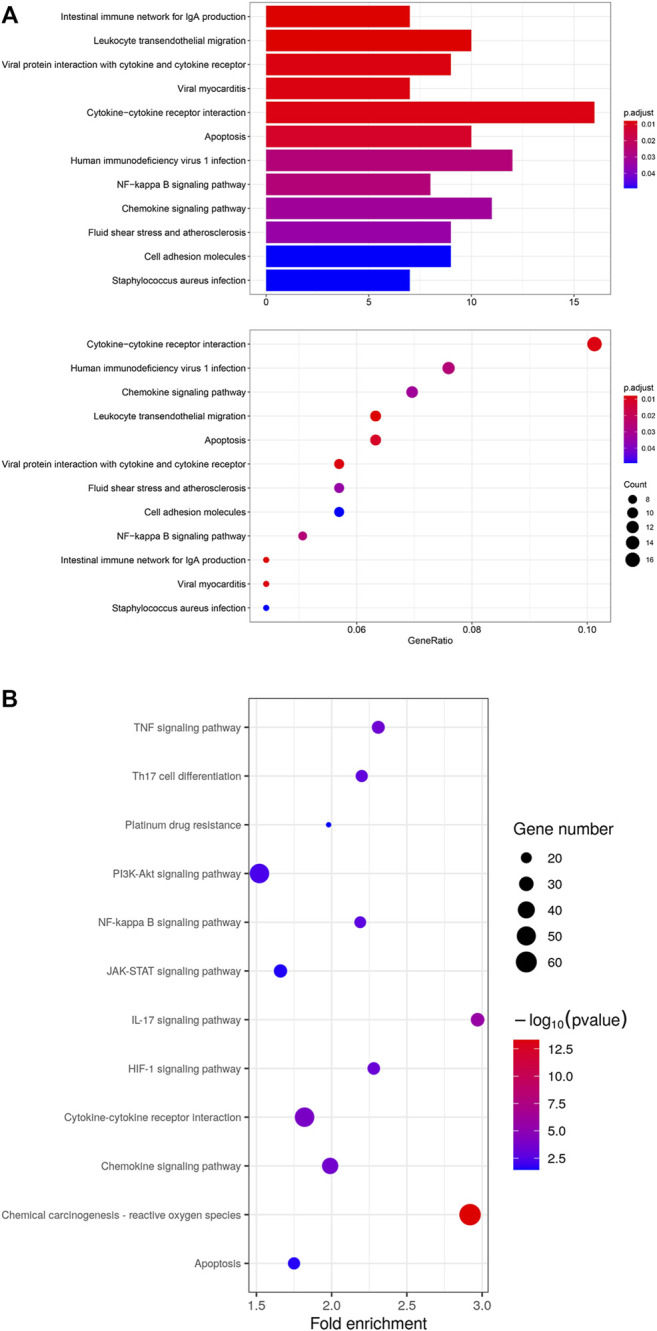
**(A)** KEGG analysis of 12 significant functional pathways in epithelial cells of residual tumor. **(B)** KEGG analysis of 12 selected pathways out of 81 significant pathways in CAFs of residual tumor.

To investigate the enriched functions of DEGs in residual tumor, signaling pathway analysis cellular subtypes was performed. The KEGG analysis revealed 12 functional pathways enriched significantly in epithelial cells. It highlighted inflammatory cytokine signaling pathways, including cytokine-cytokine receptor interaction and chemokine signaling pathways. The previous study of OSCC using scRNA-seq highlighted the “cross-talk” between cancer cells and CAFs. Thus, we investigated the functional pathways in CAFs. A total of 81 pathways were significantly enriched in CAFs, including cytokine-cytokine receptor interaction and chemokine signaling pathway, as well as signal transduction mediated via PI3K-Akt, NF-kappa B, JAK-STAT, TNF, platinum drug resistance and HIF-1. (Figure 6)

**FIGURE 7:**
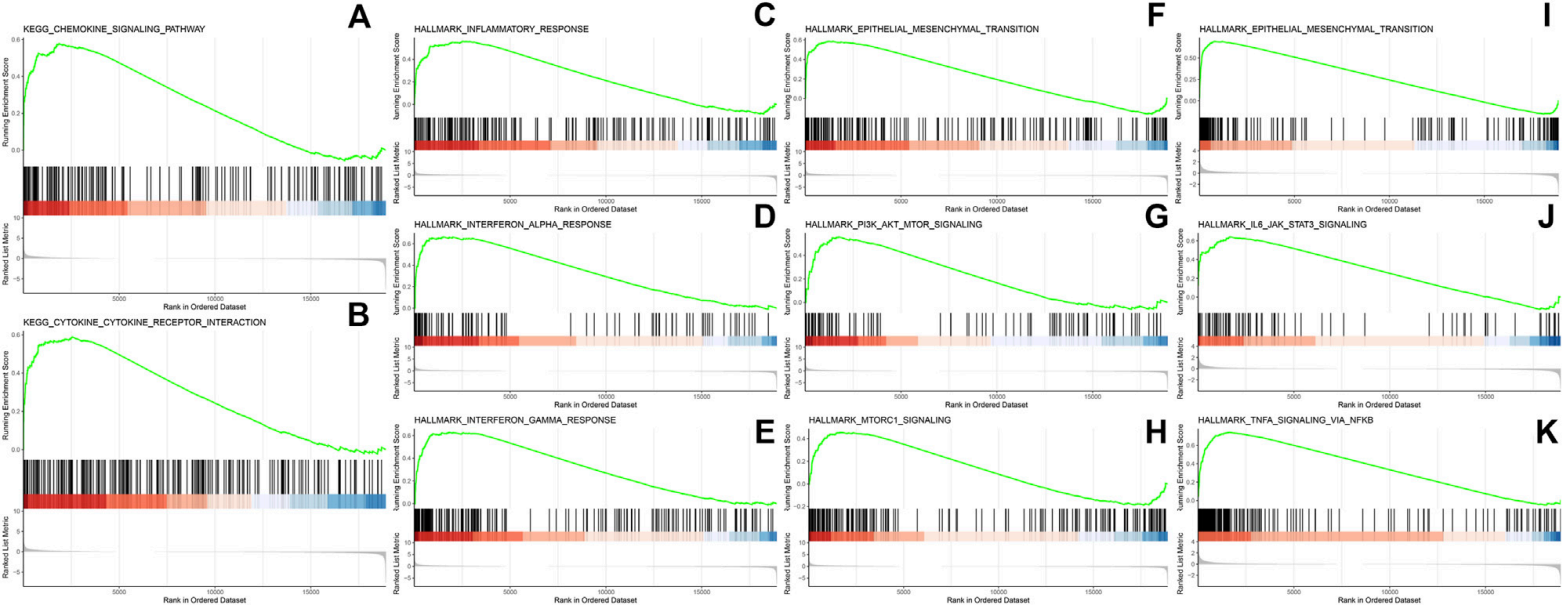
GSEA analysis in epithelial cells in residual tumors showing enriched pathways including chemokine signaling pathway **(A)**, cytokine-cytokine receptor interaction **(B)**, inflammatory response **(C)**, interferon alpha response **(D)**, interferon gamma response **(E)**, epithelial mesenchymal transition **(F)**, PI3K/AKT/MTOR signaling **(G)** and MTORC1 signaling **(H)**. GSEA analysis in CAFs in residual tumors showing enriched pathways in epithelial mesenchymal transition **(I)**, IL6/JAK/STAT3 signaling **(J)** and TNFα signaling *via* NF-κB **(K)**.

GSEA revealed 15 pathways enriched significantly in epithelial cells of residual tumor, including inflammatory response, interferon alpha and interferon gamma response, as well as PI3K/Akt/mTOR signaling and mTORC1 signaling pathways. Twenty-three pathways were enriched in CAFs, including TNFα signaling via NFκB and IL6-JAK-STAT3, IL2-STAT5, angiogenesis, hypoxia, MYC-targets_V1, and MYC targets_V2 (Figure 7).

### Cell-Cell Communications in the TME of OSCC After Chemotherapy

**FIGURE 8:**
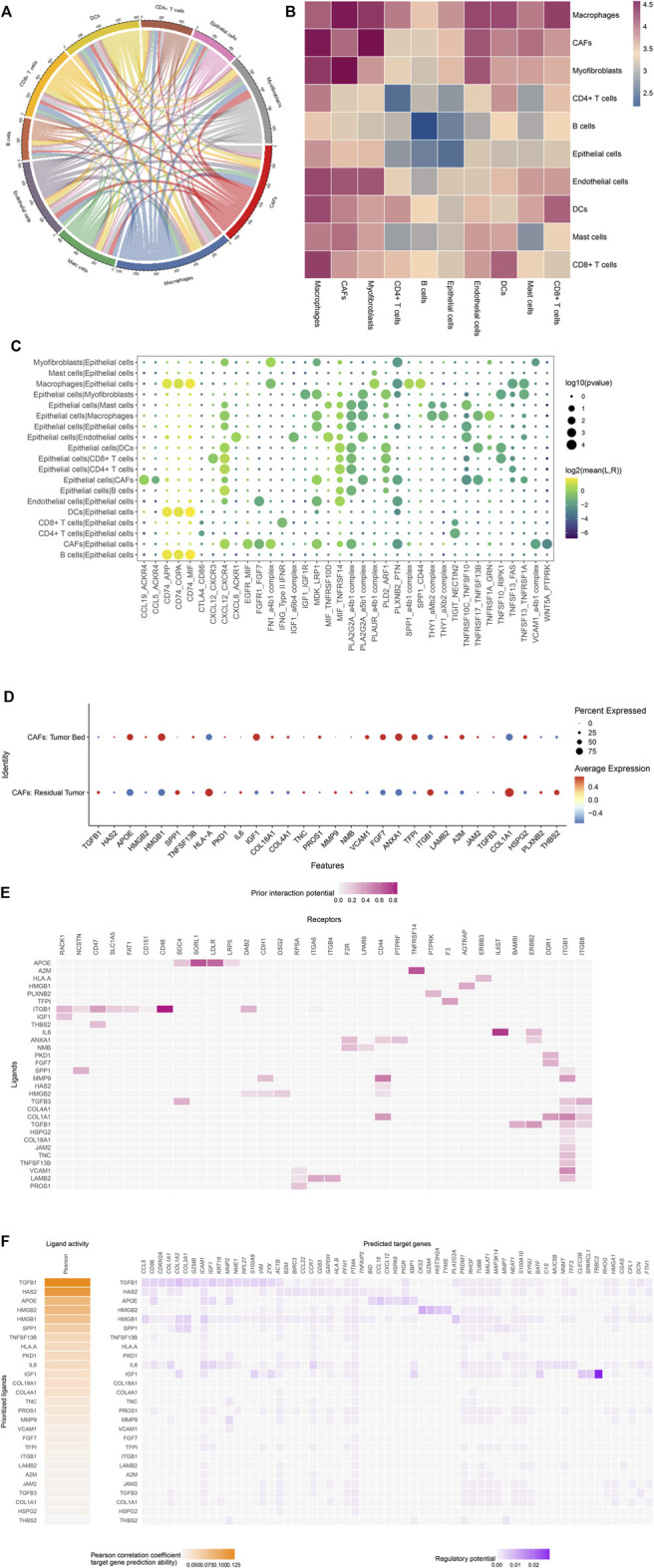
(Continued).

CellPhoneDB analysis was used to identify the molecular interactions between ligand-receptor pairs in order to construct intercellular communication networks. The interaction network and amount of potential ligand-receptor pairs among the predicted cell type in residual tumor were shown in Figures 8A,B. Based on the results of CellPhoneDB, the CD74-MIF/COPA/APP interactions between B cells, dendritic cells, macrophages and epithelial cells were identified in the residual tumor compared with tumor bed (Figure 8C). As reported previously and in the present study, we focused on the cross-talk between CAFs and residual tumor cells. The top 30 predicted ligands are shown in Figure 8D, including the highly expressed TGFB1 and IL-6 in CAFs of the residual tumor. Besides, the expressions of CXCL12-CXCR4, EGFR-MIF, FGFR1-FGR7, MIF-TNFRSF14, and WNT5A-PTPRK were predicted in the cross-talk between CAFs and tumor cells.

To further analyze the cellular crosstalk between residual tumor cells and niche cells in TME, NicheNet was used to predict the ligand-receptor pairs that potentially induce the transcriptomic changes in residual tumor cells. As CAFs as sender cells, IL-6 and TGFB1 were also listed in the top predicted ligands, which was in accordance with the results of CellPhoneDB. Based on the results of NicheNet, IL6 was expressed by CAFs. The predicted receptors in tumor cells included IL6ST and ERBB2. The TGFB1-ERBB2/ITGB1/ITGB8 and ANXA1-F2R/CD44/PTPRF/ERBB2 interactions were also predicted (Figure 8E). NicheNet analysis also predicted the target genes of the ligand-receptor interactions. The target genes of TGFB1 and IL-6 associated with EMT included VIM, COL1A1, COL1A2. The results prioritized the interaction between CAFs and ligands as well as tumor cell receptors regulating the expression of p-EMT genes (Figure 8F).

## DISCUSSION

The present study used single-cell transcriptome sequencing of residual tumor samples compared with tumor bed in an OSCC patient who underwent ICT before surgery. To the best of our knowledge, it is the first study investigating TME of OSCC after chemotherapy using scRNA-seq technology. Bulk RNA sequencing reflects the average expression of mRNA in tumor tissues, which cannot accurately assess the expression profile of specific cell type. The scRNA-seq facilitates comprehensive investigation of gene expression profile of TME with single-cell resolution. We analyzed the complex intratumor cellular structure and gene expression of different cell types in post-chemotherapy OSCC to explore the heterogeneity of TME in OSCC after chemotherapy.

The role of chemotherapy in OSCC is disputed. Several previous studies showed that chemotherapy suppresses tumor growth, but also induces pathways in tumor cells that have been shown experimentally to support tumor progression or encourage anti-tumor immune response (Edwardson et al., 2019). Our study showed different inflammatory cytokine expression and EMT in the microenvironment.

Cytokines include different families of chemokines, interleukins (ILs), adipokines, interferons (IFNs), transforming growth factors (TGFs) and tumor necrosis factors (TNFs). Prior studies have shown that several therapeutic agents used to treat cancer trigger inflammatory cytokine expression by cells of the immune system as well as tumor cells (Goldberg and Schwertfeger, 2010; Hanahan and Weinberg, 2011; Korkaya et al., 2011; Chin and Wang, 2014; Palacios-Arreola et al., 2014; Esquivel-Velazquez et al., 2015). Early in 1992, it was reported that paclitaxel treatment induced the macrophages to express TNF and IL-1 (Bogdan and Ding, 1992). Other studies revealed that this action of paclitaxel was distinct from its cytotoxic activity (Burkhart et al., 1994). Since then, chemotherapeutic drugs have been shown to induce inflammatory cytokines. For example, cisplatin triggers the expression of multiple cytokines including IL-6, CCL2, CCL5, CXCL8, BFGF, G-CSF, and VEGF in cancer cells or stromal cells of TME (Johnson-Holiday et al., 2011; Yan et al., 2014; Zhou et al., 2016). Cross-talk between TGF-β and IL-6 has also been identified in drug resistance in some cancer cells. In OSCC samples, active TGF-β signaling has been associated with drug resistance and correlated with IL-6 pathway activation, and knockdown of TGF-β increased sensitivity to cisplatin and radiotherapy (Chen et al., 2012). Resistance to cisplatin depends upon a positive feedback loop established by CXCL12 and CXCR4 (Nakamura et al., 2015). In accordance with previous studies, we found that inflammatory cytokine pathways were enriched in residual tumor cells. TGFB1, IL-6 and CXCL12 played an important role in the cell-cell communication between residual tumor cells and CAFs. Also, upregulation of cytokine expression induced by chemotherapy plays an important role in EMT (Son and Moon, 2010). The EMT patterns and significance remain unclear, especially in the era before scRNA-seq. Based on functional analysis and cell-cell interaction, the residual tumor manifested EMT induced by chemotherapy such as paclitaxel and cisplatin. EMT-activating transcription factors activate several molecular pathways, such as PI3K/Akt/mTORC1, and TNF-α via NFκB and TGF-β (Sha et al., 2021). These pathways were all enriched in the present study. It has also been reported that CAFs induce EMT via IL-6/JAK2/STAT3 pathway (Goulet et al., 2019) and CXCL12/CXCR4 axis (Zhang F. et al., 2020). EMT pathway was enriched in both epithelial cells and CAFs in the present study. The IL-6/JAK2/STAT3 pathway and CXCL12/CXCR4 interactions between tumor cells and CAFs were enriched. In this case, the tumor immune microenvironment was altered after ICT. It suggested that ICT might regulate the tumor immune microenvironment in OSCC. The mechanism of cross-talk requires further functional analysis.

We analyzed the heterogeneity of CAFs and TAMs in OSCC TME post-chemotherapy via scRNA-seq for the first time. In OSCC, partial EMT program at leading edge of tumor nest plays important roles and regulated by the TME. Investigators have found that the treatment response of ICT is often accompanied by fibrosis. The results of the present study revealed the heterogeneous of CAFs and found that different subgroups of fibroblasts located adjacent to residual tumor and in the tumor bed. The previous study of treatment naïve OSCC using scRNA-seq highlighted the function of CAFs in TME. The investigators divided fibroblasts into myofibroblasts expressing ACTA2, MYLK and MYL9, and CAFs expressing FAP, PDPN. The CAFs were further divided into CAF1 (COL1A2, THY1, VIM, CAV1, and MMP11) and CAF2 (FOS, JUN, FGF7, and TGFB2) (Puram et al., 2017). In the present study, we divided CAFs into three clusters, which were identified as iCAFs, apCAFs and myCAFs based on previous studies. CAFs express common fibroblast markers. The myCAFs showed an elevated expression of contractile components, ECM, and TGF-β response genes, while iCAFs expressed higher levels of immunomodulatory cytokines and chemokines (Wu and Swarbrick, 2021). The apCAFs expressing MHC class II and CD74 were reported in pancreatic ductal adenocarcinoma in 2019 (Elyada et al., 2019). It seems that apCAFs can cross-present antigen to T cells like professional antigen-presenting cells. It suggested that CAFs may participate in the improvement of tumor immune microenvironment after ICT. Also, IL-1 and TGFβ could induce apCAFs to undergo the process of EMT (Hanley and Thomas, 2020; Wu and Swarbrick, 2021). The myCAFs were located at the invasive tumor interface of breast tumors, whereas iCAFs were found in distal areas of the tumor stroma and associated with a higher number of lymphocytes (Wu et al., 2020). Our results were similar in that iCAFs were mainly located in tumor bed, while myCAFs were primarily detected in residual tumor. It provides new insights of TME in the region of tumor bed after ICT and the safety margin of the lesion in up-coming surgery in clinical practice. Meanwhile, the subgroups of CAFs found in OSCC after ICT can be potential therapeutic targets. It still requires further investigations in the interaction between stromal cells and both tumor cells and immune cells in TME.

In the combined data analysis, we found that the proportion of cells differed in the untreated tumor and residual tumor. The elevated proportion of macrophages might not only suggest the activation of the process of phagocytosis, but also play important role in regulation of TME. For example, specific subgroup of macrophages, such as C1Q (+) and SPP1 (+) TAMs can regulate tumor immune microenvironment via interaction with T cells and CAFs respectively, which were identified in NMF clustering of TAMs. TAMs constitute a major component of the TME, which was used to classify them into M1/M2 macrophages. In recent years, single-cell studies revealed the heterogeneity of TAMs in patients diagnosed with cancer. A mixed expression of M1 and M2 markers was observed in single-cell studies in TAMs in malignant tumors, suggesting complex TAM phenotypes in TME. Previous studies reported major subgroups of TAMs. We identified three classic subgroups of TAMs in the present study, namely C1Q (+), FCN1 (+) and SPP1(+) TAMs. The C1Q (+) TAMs recruit and regulate various T cell subsets. In contrast to tumor-preferred C1Q (+) TAMs, FCN1 (+) TAMs are enriched in tumor-adjacent tissue. FCN1(+) TAMs are considered as an intermediate stage during monocyte maturation into tumor macrophages. SPP1 (+) TAMs are tightly associated with CAFs and endothelial cells in modulating the TME (Zhang L. et al., 2020). Further studies are needed to elucidate their role in OSCC TME.

In addition to the classical molecular signaling pathways in oral cancer associated with drug resistance discussed above, other events may provide new clues to investigate the TME of OSCC after chemotherapy. CD74-MIF/COPA/APP interactions are expressed in TME of OSCC after chemotherapy. The expression of CD74-related ligand-receptor pairs have been associated with immune checkpoint inhibitors in a recent study using scRNA-seq. In the study, CD74-MIF/COPA/APP interactions between B cells/DCs/macrophages were downregulated in pretreatment responders and upregulated in non-responders following anti-PD-1 therapy (Jiang et al., 2021). Also, CD74 promotes tumor cell survival by interacting with macrophage migration inhibitory factor (MIF) (Tanese et al., 2015). Inhibition of CD74-MIF signaling in macrophages and DCs restores the antitumor immune response of macrophages and DCs and subsequently improves the antitumor reaction of cytotoxic T cells (Figueiredo et al., 2018). It suggests that CD74-MIF/COPA/APP interactions play an important role in TME associated with response to immunotherapy. However, few previous studies have elucidated the anti-tumor effects of these interactions, suggesting the need for further investigation.

The present study has some limitations. The challenges associated with sample collection resulted in the study investigating a single patient with partial release after ICT using scRNA-seq. Due to the limited number of samples, a further study with more number of samples is required to demonstrate the OSCC heterogeneity. The functions and signaling pathways identified in the present study warrant further investigation.

## Conclusion

In conclusion, this study revealed the characteristics of post-chemotherapy OSCC at single-cell transcriptome level. The results provide new insights into TME and clues for further investigations and treatment of OSCC.

## Data Availability

The raw data supporting the conclusion of this article will be made available by the authors, without undue reservation.

## Abbreviations

apCAFs, antigen- presenting CAFs; CAFs, Cancer-associated fibroblasts; DEGs, differentially expressed genes; EMT, epithelial-mesenchymal transition; FDCs, follicle dendritic cells; GSEA, gene set enrichment analysis; HNSCC, head and neck squamous cell carcinoma; iCAFs, inflammatory CAFs; ICT, induction chemotherapy; KEGG, Kyoto Encyclopedia of Genes and Genomes; myCAFs, myofibroblastic CAFs; NMF, non-negative matrix factorization; OSCC, oral squamous cell carcinoma; PCA, principal component analysis; QC, quality control; scRNA-seq, single-cell RNA sequencing; TME, tumor microenvironment; UMI, unique molecular identifier.
